# Announcement: First COST European Workshop on Biocoordination Chemistry

**DOI:** 10.1155/MBD.1994.341

**Published:** 1994


					VoL 1, No. 4, 1994 Announcements
FIRST C0ST
EUROPEAN WORKSHOP ON
BIOCOORDINATION CHEMISTRY
September 5-6, 1994,
Budapest, Hungary
Organizer
Central Research Institute for Chemistry of the Hungarian Academy of Sciences, Budapest
Sponsors
National Committee for Technological Development
Commission of the European Communities
COST Secretariat Chemistry
Local Organizing Committee
L.I. Simandi (Chairman) T. Bama
T. Kiss B. Noszal
E. BrOcher
I. Sovago
K. Burger
G. Speier
International Advisory Board
J. Reedijk (Chairman)
On behalf of the D1 Management Committee
Venue
Central Research Institute for Chemistry, Budapest
H-1025 Budapest, Pusztaszeri Ot 59/67, Hungary
Scope
All aspects of biocoordination chemistry, including structural, equilibrium and
kinetic studies on
-metal complexes of bioligands (amino acids, peptides, DNA, proteins,
nucleobases, porphyrins, carbohydrates, etc.)
metalloenzymes
-enzyme modelling and related catalytic aspects
activation of small molecules (02, H2, etc.)
-metal complexes in cancer chemotherapy
NMR imaging.
Special emphasis is placed on
supported by COST Action D1,
projects
341
VoL I, No. 4, 1994 Announoements
Main Speakers
Jan Reedijk (Leiden): Recent developments in metaI-DNA binding
Geoffrey Sykes (Newcastle)' Mechanistic studies on the tyrosyl radical Fe(lll)2
and containing enzyme ribonucleotide reductase
Giovanni Natile (Bari): Platinum based anticancer agents: new structures for
different mechanisms
Erik Larsen (Copenhagen): Activation of a C-II bond by coordination of an ylid
Kalmdn Burger (Szeged) Metal ion coordination of bioactive ligands
Thomas Kaden (Basel) Homoditopic and heteroditopic bismacrocycles and their
metal complexes
Ern6 Br0cher, Robert Kirdly, iva Tth and Imre Tth (Debrecen)
Complexation properties of some macrocyclic tetraazapolyacetate ligands of
biological interest
Hugh D. Powell and Andr E. Merbach (Lausanne)" Relevance of high
pressure kinetic studies to the design of better MRI contrast agents
Laszlo I. Simdndi (Budapest)' Free radical intermediates in cobaloxime
catalyzed oxidations
Imre Svdgd (Debrecen)" Eqilibrium and structural studies on transition metal
peptide complexes
A, Kirsch-de Mesmaeker (Bruxelles)" Photoreactions of polyazaaromatic Ru(ll)
comp]exes on targeted DNA sites
M. Leng (CNRS, Orleans)" to be announced
Edward Szlyk (Torun)" Adenosine coordination compounds of nickel(ll) and
zinc(ll)
M. Schroeder (Edinburgh)" Towards biocoordination with macrocylic complexes
John M. Kelly (Dublin): Photoadducts of ruthenium(ll)-tris-l,4,5,8-
tetraazaphenanthrene with guanine and DNA
Tarbj6rn Drakenberg (Espoo, Finland): Structural and dynamic effects of
calcium binding to a mutant cellbinding Dgk
Lucio Randaccio (Trieste): Structure and bonding in cytosine nucleobase
complexes with metal-metal bonds
Additional information can be obtained from"
Prof.
LR.I. Simandi
Central esearch Institute for Chemistr
of the Hungarian Academy of Sciences
H-1525 Budapest, Pf. 17 Hungary
Fax: (36-1) 135-2112
342

				

